# Non-canonical capsid engineering highlights new possibilities for AAV vectorology

**DOI:** 10.1016/j.omtm.2024.101221

**Published:** 2024-03-06

**Authors:** Zehan Zhang, John R. Counsell

**Affiliations:** 1 Research Department of Targeted Intervention, UCL Division of Surgery and Interventional Science, Charles Bell House, London, UK

## Main text

As the first wave of gene therapies have passed through initial first-in-human trials, they have highlighted a need to develop targeted delivery vectors with enhanced transduction profiles. Within this landscape, recombinant adeno-associated virus (rAAV) remains one of the most popular and versatile gene delivery vectors. The majority of AAV clinical trials have focused on use of naturally occurring 1, 2, 5, 8, and 9.^1^

Over the past decade, increasing efforts have been made to confer greater precision and efficacy in cell targeting via capsid engineering, which can be mediated via a number of mechanisms.^2^ This has unearthed a plethora of novel capsids with enhanced functionality for a variety of gene therapy applications, which may lead to improved long-term clinical and commercial prospects for AAV therapies.

In the described paper, Chang and colleagues report potential benefits of incorporating non-canonical amino acids (ncAAs) into AAV capsid proteins to modify their activity. This approach is potentially interesting for AAV capsid engineering considering that the physicochemical properties of peptide sequences could be expanded beyond what is possible with the standard set of 20 amino acids, thereby presenting an alternative strategy to modulate interactions made by capsid domains of interest.

**Figure 1:**
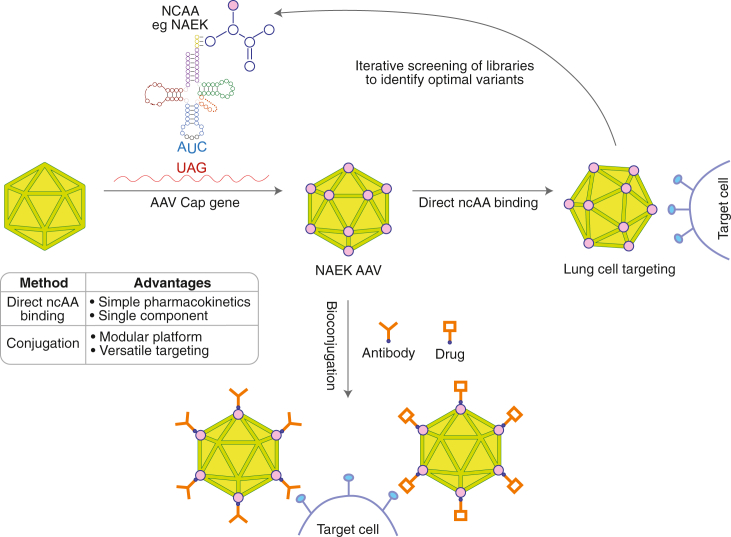
Schematic describing applications of AAV engineering with ncAAs, including that described by Chang et al., versus historical engineering strategies focused on bioconjugation

In this study, the authors incorporated Nε-2-azideoethyloxycarbonyl-L-lysine (NAEK) into AAV5 capsid variable regions via genetic code expansion technology (Figure 1).^3^ The NAEK-AAV5 vector demonstrated enhanced transduction of a variety of cell types versus parental AAV5 while exhibiting a lung-specific transduction profile *in vivo*. This highlighted, for the first time, that direct incorporation of ncAAs into AAV5 capsids could offer significant potential as a standalone modification. This is in contrast to the majority of previous studies, which have employed ncAAs as “molecular handles” to facilitate the development of antibody-drug conjugates through click chemistry reactions, utilizing biorthogonal functional group such as azides.^4^^,^^5^^,^^6^

In the field of AAV engineering, this has been applied primarily to AAV2 capsids to modify their targeting properties via antibody conjugation.^7^^,^^8^^,^^9^^,^^10^ The work of Chang et al. has demonstrated that direct ncAA incorporation alone has the potential to significantly enhance vector transduction, which resonates with previous research described by Horowitz et al., where non-canonical side chains were introduced chemically via an exogenous glycation reaction on capsid arginines.^11^

ncAA-engineered capsids could overcome several limitations faced by those that depend on antibody conjugates, such as complex pharmacokinetics, inconsistent conjugated final product, and additional purification steps post-conjugation, which may require process research to overcome potential manufacturing and quality hurdles. Indeed, even the approach described in this paper was affected by perceived inconsistency with NAEK incorporation into the capsid, which would likely require addressing in further development.

One of the variants developed in this study (374NAEK-AAV5) showed 2.5- to 10.5-fold increased transduction efficiency over parental rAAV5 when tested *in vitro* across various cell lines. Subsequent *in vivo* investigations with a dose of 1 × 10^11^ vector genomes revealed enhanced lung-specific transduction, especially to alveolar type 2 cells, via both systemic and intranasal delivery. Interestingly, 374NAEK-AAV5 showed higher lung transduction efficiency when compared to the comparator serotypes AAV6, AAV6.2, and AAV6.2FF. However, one advises caution when drawing conclusions on specificity given that the physicochemical properties of NAEK side chains could theoretically tether the virus indiscriminately, leading to increased but non-specific transduction.

The authors did address this possibility by delving deeper into the potential binding mechanism. As indicated, 374NAEK was in proximity to 498E in the neighboring beta turn, potentially interacting via hydrogen bonds. This suggested the possibility of either a new binding pocket being created or an alteration in the structure of 3-fold axis, possibly leading to lung specificity. Nevertheless, further validation of this hypothesis through cryoelectron microscopy is necessary to identify the exact target of 374NAEK-AAV5 and the binding model to substantiate the observed lung targeting mechanism.

In summary, Chang et al. have highlighted the possibility of generating novel AAV capsids with enhanced functionality by incorporating ncAAs without additional conjugation of ligands or antibodies. Expanding on this study, innovative approaches may look at combining this approach with high-throughput screening strategies to select ncAA-engineered capsid variants with desired properties, further expanding the pool of available gene therapy vector technologies.
